# 在YUV色彩空间中自荧光气管镜图像定量方法的临床应用

**DOI:** 10.3779/j.issn.1009-3419.2014.11.05

**Published:** 2014-11-20

**Authors:** 筱轩 郑, 红凯 熊, 勇 李, 宝惠 韩, 加源 孙

**Affiliations:** 1 200030 上海，上海交通大学附属胸科医院内镜室 Department of Endoscopy Room, Shanghai Chest Hospital, Shanghai Jiao Tong University, Shanghai 200030, China; 2 200240 上海，上海交通大学电子信息与电气工程学院 Department of Electronic Engineering, Shanghai Jiao Tong University, Shanghai 200240, China; 3 200030 上海，上海交通大学附属胸科医院肺内科 Department of Pulmonary Medicine, Shanghai Chest Hospital, Shanghai Jiao Tong University, Shanghai 200030, China

**Keywords:** 肺肿瘤, 诊断, 自荧光气管镜, 白光气管镜, 医学图像处理, Lung neoplasms, Diagnosis, Autofluorescence bronchoscopy, White-light bronchoscopy, Medical image processing

## Abstract

**背景与目的:**

通过对不同病理类型的自荧光气管镜（autofluorescence bronchoscope, AFB）图像目标区域的YUV定量分析，确定区分不同疾病类型的最佳判别指标，探讨AFB在中央型支气管肺癌诊断中的价值。

**方法:**

对研究对象进行白光气管镜+AFB检查，二者在镜下存在异常者行活检。并对荧光图像显示病变部位通过MATLAB图像测量软件进行YUV定量分析。根据正常支气管粘膜、炎症、低级别上皮样瘤变、高级别上皮样瘤变、浸润性癌的病理结果分组。研究各组与YUV值间的关系，所得数据采用SPSS 11.5软件进行统计学处理。

**结果:**

Y值在浸润性癌和LGD组间存在统计学差异（*P*=0.040），在浸润性癌和炎症组也存在明显统计学差异（*P* < 0.001）。其他的各组间无统计学差异。U值在浸润性癌和HGD、LGD、炎症、正常支气管粘膜组之间存在统计学差异（*P* < 0.050），能较好鉴别正常粘膜及恶性病变。V值在浸润性癌和LGD组（*P*=0.003）、炎症组（*P* < 0.001）、正常支气管粘膜组（*P* < 0.001）存在统计学差异，能有效鉴别浸润性癌及良性疾病。V值在正常支气管粘膜组与HGD组（*P*=0.001）、炎症组（*P*=0.004）间比较也具有统计学差异。

**结论:**

利用YUV色彩空间系统针对支气管和肺良恶性疾病鉴别有一定临床应用价值，为临床气管镜诊断肺癌及癌前病变提供有效科学依据。

## Abstract (en)

**Background and objective:**

The aim of this study is to determine the best reference values of the optimal evaluation indexes that identify different disease types. Disease identification was conducted using the YUV quantitative analysis of autofluorescence bronchoscopy (AFB) images in the target areas. Furthermore, this study discusses the significance of AFB in the diagnosis of the central-type lung cancer.

**Methods:**

A biopsy was conducted for cases that showed pathologic changes under either autofluorescence or white-light bronchoscopy. Moreover, MATLAB was used to carry out the quantitative analyses of lesion in multi-color spaces from AFB images. The cases were divided into different groups according to the pathological diagnosis of normal bronchial mucosa, inflammation, low-grade dysplasia (LGD), high-grade dysplasia (HGD), and invasive cancer. SPSS 11.5 was used to process the data for statistical analysis.

**Results:**

The Y values were different and statistically different between invasive cancer and LGD (*P* < 0.001) and invasive cancer and inflammation (*P*=0.040), respectively. The U values between invasive cancer and the other groups were statistically different (*P* < 0.050). Similarly, the V values between invasive cancer and LGD and inflammation and normal bronchial mucosa were different. Lastly, the V values between normal bronchial mucosa and HGD and inflammation and normal bronchial mucosa were different.

**Conclusion:**

The YUV values in the AFB effectively identified benign and malignant diseases and were proven to be effective scientific bases for the accurate AFB diagnosis of lung cancer.

肺癌是全球最常见的恶性肿瘤之一，也是当前我国乃至全球肿瘤死亡的主要原因，肺癌的发病率和死亡率位居癌症首位。肺癌的预后与肿瘤分期密切相关，早期肺癌的5年生存率比晚期肺癌的5年生存率明显提高^[1-3]^。因此，如何早期发现、早期诊断、早期治疗是改善肺癌患者预后的关键。

支气管镜检查和痰细胞学检查是目前临床上发现早期中央型肺癌（central type early lung cancer, CELC）的主要方法，痰细胞学检查阳性率很低，支气管镜检查也仅在一定程度上增加CELC的发现率^[4]^，但多数的CELC仅显示轻微的支气管粘膜的改变，使用普通光源支气管镜检查（white light bronchoscopy, WLB），即使有经验的医生也很难发现侵袭前损害，而自荧光气管镜在一定程度上得到了改进，弥补了不足^[5]^。因此，我们迫切的需要寻找新的检查手段来早期发现、早期定位及诊断肺癌，从而改善患者预后，降低肺癌的死亡率。

自荧光气管镜检查是利用细胞自发性荧光和电脑图像分析技术开发的一种新型支气管镜，可使气管镜对肺癌及其癌前病变早期定位诊断的灵敏度提高，是对传统WLB的技术突破。目前AFB检查也存在一定的局限性，如特异度不强，在支气管粘膜炎症、炎性肉芽肿、瘢痕组织、粘膜损伤等情况下，局部也会表现为红色荧光，极易与癌前病变、原位癌、浸润癌相混淆。且目前仅凭色彩改变而定性进行结果判断的诊断方法存在较大的局限性，色彩信息的复杂性仅凭肉眼观察定性诊断不利于实践操作中的精准判断。

因此，我们希望通过对色彩的分析，将色彩信息量化，得到更直观的数字信息来更准确的指导临床工作，通过对肺癌发展过程中不同组织病理阶段荧光强度的量化，使其在肺癌的早期诊断、评估局部癌变的程度中发挥更大的价值。

## 1 资料与方法

### 1.1 临床资料和方法

回顾性地分析了上海市胸科医院2012年4月-2014年1月住院及门诊患者使用自荧光图像（autofluorescence image, AFI）系统的荧光支气管镜及白光支气管镜进行检查的图像信息，用量化的方法对AFB在肺癌诊断及鉴别诊断中的应用价值进行初步探索及研究。

### 1.2 入组病例基本情况

共入组有效病例218例，均进行WLB和AFB检查。其中男性186例，女性32例，平均年龄59.9岁（22岁-84岁）。所有患者均在上海市胸科医院气管镜室进行检查。检查前所有患者均被告知气管镜检查风险并签署知情同意书。

### 1.3 入选标准

新近出现的或原有症状性质发生改变，如咳嗽、咳痰、咯血、声嘶、体重减轻等，临床怀疑肺癌，痰细胞学检查发现异常，影像学检查提示肺部占位、肺不张或片状阴影等怀疑中央型肺癌患者。

### 1.4 排除标准

有气管镜检查病史且已行活检、刷检，有气管镜检查禁忌证（活动性大咯血、不稳定心绞痛、麻醉药过敏等），6个月内进行过细胞毒药物化疗，3个月内服用致光敏药物。

### 1.5 气管镜操作方法

#### 1.5.1 术前准备和麻醉

术前禁食、水4 h以上，和患者及家属沟通，让其做好心理准备，以便能更好的配合检查。局部麻醉：检查前采用2%利多卡因经口滴注，加用7%利多卡因进行喉部深部喷雾3喷-5喷，检查过程中经声门、气管、左右总支气管常规予以2%利多卡因喷洒。整个操作过程均予以心电监护，监测血压、心率、血氧饱和度，并鼻导管吸氧。

#### 1.5.2 气管镜检查方法

经口腔置入BF-F260型自荧光气管镜（日本，奥林巴斯公司），首先在普通白光状态下检查声门、气管、隆突、左右主支气管及各个叶、段、亚段支气管，观察软骨环、粘膜、血管、分泌物和新生物等，并清除气道内分泌物，记录所有可疑的病变情况，包括粘膜充血、水肿、增厚、结节、血管聚拢、颗粒状突起、支气管间嵴增宽等，并采集图像。然后切换至荧光状态，对气道重新检查，对白光状态下的可疑部位进行重点检查，并在距离病灶1 cm-3 cm距离做图像采集等详细记录，全部气道检查完毕后，对每一处可疑部位进行活检、刷检、冲洗等细胞学及病理检查。

#### 1.5.3 气管镜检查结果镜下评判

普通白光状态下可见病变分为三级^[6]^：WLBⅠ：先天解剖异常、外压性病变、单纯支气管间嵴增宽、粘膜色泽正常、不伴有充血水肿；WLBⅡ：粘膜充血、水肿、增厚、色泽改变、血管聚集或扭曲；WLBⅢ：粘膜颗粒样改变或明显新生物

将WLBⅡ和WLBⅢ归为WLB检查的异常表现。荧光状态下可见病变同样分为三级：AFBⅠ：粘膜绿色；AFBⅡ：粘膜色泽轻度改变，淡粉色或棕色；AFBⅢ：粘膜变成典型的红色或紫红色。

将AFBⅡ和AFBⅢ定义为AFB异常表现。WLB及AFB检查均由两位经验丰富的医师进行检查，两位操作医师在不知晓病理结果的情况下分别对图像进行评级，若评级不相同由主任医师加入后讨论决定评级。

### 1.6 荧光图像的分析及处理

若荧光图像中病灶覆盖有分泌物，则予以剔除，若病灶中存在少许出血点，则不作为靶区选择，若病灶中出血范围较大则予以剔除。图像采集均由同一套系统采集完成并储存，由同一台电脑内的MATLAB（R2012b版本，MathWorks公司，美国）软件进行处理，测量病灶中央一处及边缘两处16×16像素靶区，并取三处数值的平均值为最终数据，通过软件分析得出YUV值。

YUV色彩空间是一种图像分析的常用方法。其中Y指颜色的明视度，即亮度，其实Y就是图像的灰度值；而U和V分量通常被并称为色度，用于表示图像的色调和饱和度，色调信息主要是反映颜色的类别，它决定了颜色的基本特征；而饱和度反映的是某一种颜色的纯度，通俗来讲就是颜色的深浅程度。YUV颜色空间最大的优点就是，亮度信号（Y）和色度信号（U, V）是相互独立的。

### 1.7 病理结果判断

病理诊断由两位经验丰富的病理科医生担任，在不知晓操作医生分级的情况下对病理作出诊断，若两位医生诊断不同，则由主任医师加入讨论后做出诊断。讨论后得出的结论作为最终结论。

将病理诊断分为浸润性癌（invasive cacinoma）、原位癌（carcinoma *in situ*, CIS），重度不典型增生（severe dysplasia）、中度不典型增生（moderate dysplasia）、轻度不典型增生（mild dysplasia）、增生（hyperplasia）、鳞状上皮化生（squamous metaplasia）、炎症（inflammation）、正常（normal cell）^[7]^。

将病理诊断为重度不典型增生及原位癌定义为高级别上皮内瘤变（high-grade preinvasive, HGD）；增生、鳞状上皮化生、轻、中度不典型增生定义为低级别上皮内瘤变（low-grade preinvasive, LGD）。并将病理诊断为HGD、浸润性癌定义为阳性诊断；正常粘膜、炎症、LGD定义为阴性诊断^[6]^。

### 1.8 统计学方法

采用SPSS 11.5软件进行数据处理，计量资料以均数±标准差（Mean±SD）表示，计数资料以百分率表示。组间均数比较采用单因素方差分析及LSD方法检验。以*P*＜0.05为差异有统计学意义。

## 2 结果

### 2.1 病理检查结果

所有患者气管镜检查顺利，检查过程中未出现严重并发症。病例标本中特异性感染如真菌感染，结核等，予以剔除后，共获得218例有效病理标本。

218例有效病例中鳞癌72例，腺癌31例，小细胞癌31例，低分化癌及未分型癌16例，急慢性炎症或炎细胞浸润32例，重度不典型增生及原位癌14例，LGD 9例，正常支气管粘膜上皮13例（表 1）。

**1:** 气管镜活检病理诊断 Bronchoscopy biopsy diagnosis

Pathological diagnosis	Number of cases (*n*)	Percentage (%)
Squamous cell carcinoma	72	33.0
Adenocarcinoma	31	14.2
Small cell carcinoma	31	14.2
Undifferentiated carcinoma	16	7.3
HGD	14	6.4
LGD	9	4.1
Normal	13	6.0
Inflammation	32	14.7
Total	218	100

### 2.2 病理诊断与荧光图像定量值（YUV系统）之间关系

在218例有效病例中，YUV值在各病理组间的均数及标准差（表 2）。

**2:** YUV值 YUV values

Group	*n*	Y	U	V
Invasive cancer	150	0.43±0.14	-0.00±0.03	0.15±0.06
HGD	14	0.45±0.16	-0.02±0.02	0.12±0.06
LGD	9	0.54±0.18	-0.04±0.02	0.08±0.03
Inflammation	32	0.53±0.18	-0.04±0.04	0.10±0.08
Normal	13	0.51±0.20	-0.07±0.03	0.05±0.03

#### 2.2.1 Y值与各病理组的相关性

Y值在浸润性癌、HGD、LGD、炎症、正常支气管粘膜组的均值及标准差分别为0.43±0.14、0.45±0.16、0.54±0.18、0.53±0.18、0.51±0.20（图 1）。

**1:**
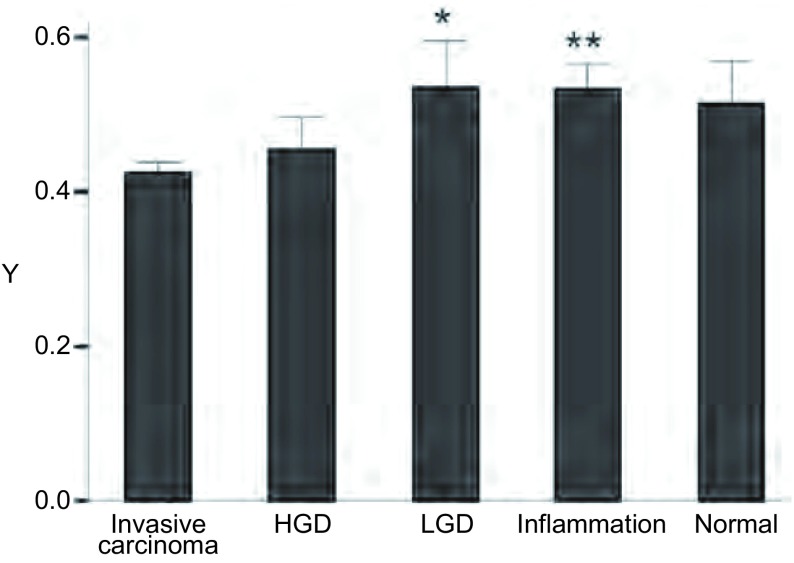
荧光气管镜图像Y值 Y value in AFB. ^*^: compare with invasive carcinoma, *P* < 0.05; ^**^: compare with invasive carcinoma, *P* < 0.01. HGD: high-grade preinvasive; LGD: low-grade preinvasive; AFB: autofluorescence bronchoscope.

Y值在浸润性癌和LGD组间存在差异（*P*=0.040），Y值在浸润性癌和炎症组也存在差异（*P*＜0.001）。其他的各组间无统计学意义。

结果显示Y值在个别组间差异有统计学意义，但不能明显区分良恶性疾病或癌前病变与浸润癌，可能无明显实际临床意义。

#### 2.2.2 U值与各病理组的相关性

U值在浸润性癌、HGD、LGD、炎症、正常支气管粘膜组的均值及标准差分别为-0.00±0.03、-0.02±0.02、-0.04±0.02、-0.04±0.04、-0.07±0.03。荧光气管镜图像在各组间的U值（图 2）。

**2:**
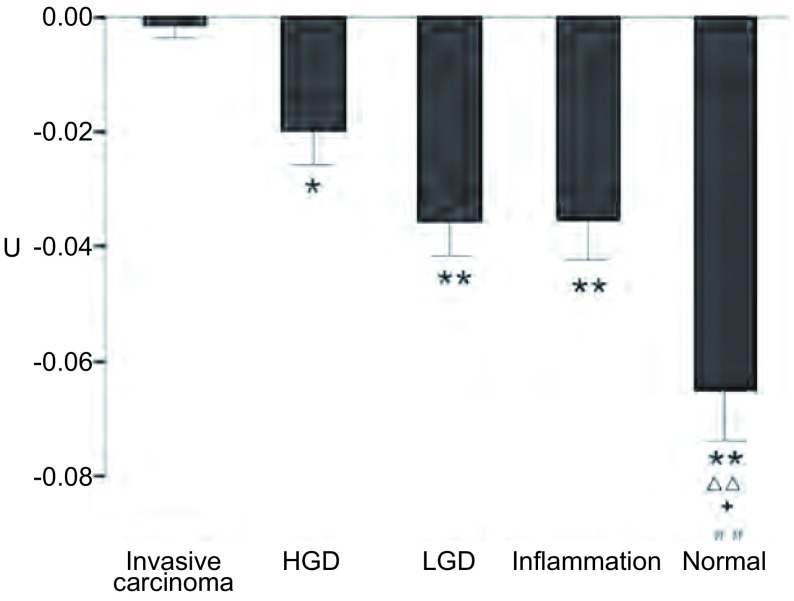
荧光气管镜图像U值 U value in AFB. ^*^: compare with invasive carcinoma, *P* < 0.05; ^**^: compare with invasive carcinoma, *P* < 0.01; △△: compare with HGD, *P* < 0.01; +: compare with LGD, *P* < 0.05; ##: compare with inflammation, *P* < 0.01.

U值在浸润性癌和HGD组间存在统计学差异（*P*=0.025），且浸润性癌和LGD组（*P*=0.001）、炎症组（*P*=0.001）及正常支气管粘膜组（*P*＜0.001）均存在明显差异（*P*＜0.010）。

正常支气管粘膜组与HGD组（*P*＜0.001），炎症组（*P*=0.002）存在差异。正常支气管粘膜组与LGD组也差异有统计学意义（*P*=0.020）。

结果显示U值能较好的区分浸润性癌与其他组别，并能较好的区分正常支气管粘膜与其他组别，但在HGD、LGD、炎症组间区别意义不大。

#### 2.2.3 V值与各病理组的相关性

V值在浸润性癌、HGD、LGD、炎症、正常支气管粘膜组的均值及标准差分别为0.15±0.06、0.12±0.06、0.08±0.03、0.10±0.08、0.05±0.03。荧光气管镜图像在各病理组间的V值（如图 3)。

**3:**
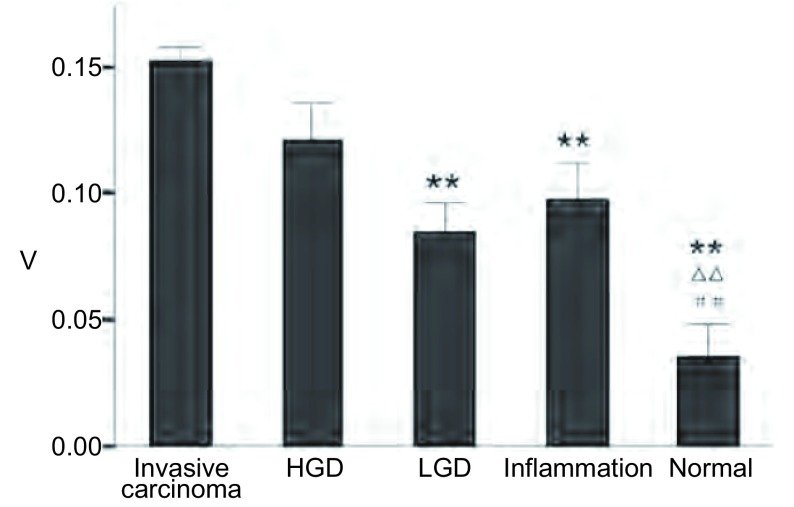
荧光气管镜图像V值 V value in AFB. ^**^: compare with invasive carcinoma, *P* < 0.01; △△: compare with HGD, *P* < 0.01; ##: compare with inflammation, *P* < 0.01.

V值在浸润性癌和LGD组（*P*=0.003）、炎症组（*P*＜0.001）、正常支气管粘膜组（*P*＜0.001）存在差异。V值在正常支气管粘膜组与HGD组（*P*=0.001）、炎症组（*P*=0.004）间比较也具有差异。

结果显示V值在部分组间存在差异，在疾病的鉴别诊断中有一定意义。

### 2.3 U值ROC曲线

U值能较好的鉴别癌前病变及浸润性癌，根据研究所得数据作关于鉴别病理诊断为浸润性癌及HGD的ROC曲线，曲线下面积达0.697（图 4）。U值取-0.01227作为cut off值，则诊断灵敏度达65.3%，特异度达58.1%。因此，可用该指标作为评价标准应用于临床荧光气管镜操作中以鉴别癌前病变与浸润性癌。

**4:**
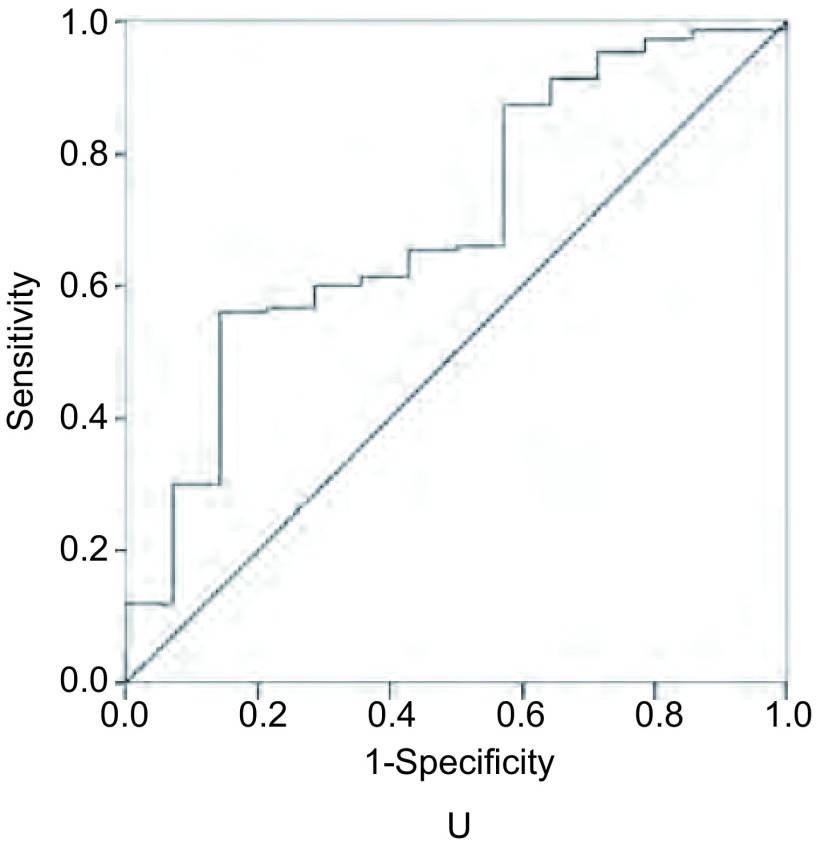
U值ROC曲线（鉴别HGD和浸润性癌） ROC curve of U value for detection of HGD and invasive cancer

## 3 讨论

肺癌已成为导致癌症病例死亡的最常见恶性肿瘤之一，由于吸烟、空气污染等多方面因素，其死亡率仍逐年上升，且易发生脑转移^[8]^。对肺癌早期诊断、早期治疗将有效降低死亡率。肺癌的早期诊断方法主要有细胞病理学、影像学、纤维支气管镜等技术。在早期肺癌中央型和外周型两大类中，胸部螺旋CT对于外周性病灶的灵敏度较高^[9, 10]^。而常规白光气管镜对早期中央型肺癌诊断有一定帮助，但之后出现的自荧光气管镜能更好的发现早期支气管肺癌^[11-15]^。

目前国外多数研究报道AFB单用或联合WLB使用，在检测癌前病变和癌变组织方面的灵敏度均比单用WLB高^[14]^。Mssaki Hanibuchi等^[15]^研究报道了AFB（SAFE-1000系统）在检测癌前病变和癌变组织中的价值，WLB+AFB联合应用的灵敏度为96.8%，明显高于单纯WLB的灵敏度65%。另外，Ikeda等^[16]^研究报道显示，在发现不典型增生病变及原位癌方面，WLB的灵敏度为65%，而AFB（SAFE-3000系统）的灵敏度达90%。稍后出现的AFI系统，在日本、加拿大、美国及欧洲国家等都曾有相关研究；多中心和随机对照研究已经证实了AFB联合WLB发现中重度不典型增生和原位癌的优势。WLB联用AFB后，相对WLB检查，中度至重度不典型增生和原位癌的诊断灵敏度可提高1.4倍、3.7倍、4.3倍、5.8倍、6.3倍不等^[17-20]^。但某些因素如：气管镜检查过程中对气道壁的摩擦损伤、气道粘膜炎症、口服抗凝药物、3个月内服用致光敏药物、6个月内进行过细胞毒药物化疗等可使荧光支气管镜下出现假阳性结果^[9]^。此外，由于AFB检测不仅在癌灶中呈阳性改变，在炎症等良性病变中也呈阳性表现，因此特异度较差，因此在一定程度上降低了其在气道病变的临床应用价值。

本研究探索性的将自荧光气管镜图像运用于色彩空间中进行分析研究，使图像信息量化，更利于临床实际操作。在色彩定量分析系统中RGB色彩空间是目前运用最广的颜色系统之一，即是代表红、绿、蓝三个通道的颜色，这个标准几乎包括了人类视力所能感知的所有颜色。然而，在处理现实世界的图像时RGB并非很有效，而且处理RGB色彩空间的图像也利用了较多的运算空间，转化速度较慢。由于上述和其他一些原因，许多广播、电视系统、成像标准使用亮度和色差视频信号，即YUV色彩空间。可以说在计算机图像处理领域，YUV色彩空间同样占据着重要地位^[21]^。因此，本研究利用自荧光气管镜图像在YVU系统中的不同表现进行统计分析研究。

在本研究中，我们利用自荧光气管镜图像YUV色彩空间定量值进行处理，测量病变区域的YUV值，由于自荧光气管镜图像的色彩在各病理诊断组间存在差异，统计结果显示U值在浸润性癌和HGD、LGD、正常支气管粘膜、炎症组存在差异，U值在正常组别中最低，而在浸润性癌组别中最高。其量化的指标能更客观准确的指导临床镜检。利用客观定量YUV值判断靶区病变性质，有利于患者疾病的诊断及医师临床气管镜的操作，避免了主观因素的干扰。U值在荧光图像中能较好的鉴别良恶性疾病。在浸润性癌与HGD对比中取-0.012, 27为cut off值，则灵敏度达65.3%，特异度达58.1%，曲线下面积达0.697。因此，若将该cut off值作为评判良恶疾病的指标，将有效提高自荧光气管镜诊断的阳性率，指导临床操作，避免其他因素影响。

AFB的缺点在于其特异度较低，易受气道炎症、充血、出血、支气管粘膜损伤等因素的影响，假阳性率较高。而本研究通过各种病理类型疾病荧光图像的色度差异，利用荧光图像定量方法有效区分炎症等良性疾病与恶性疾病，可进一步提高肺癌特别是早期肺癌的诊断率，提高诊断的特异度，提高患者5年生存率。随着AFB荧光图像定量方法的推广及科学技术的发展，将对肺癌发展过程中不同组织病理阶段荧光强度进行量化，使AFB在肺癌的早期诊断、明确病变范围、评估局部癌变程度的过程中发挥更大的作用。

相信通过对AFB激发光源、图像处理的深入研究及对成像系统的进一步改善，AFB将在探查和诊断肺部疾病方面具有更重要的应用价值及临床意义。

## References

[1] Shiono S, Abiko M, Sato T (2013). Limited resection for clinical stage IA non-small-cell lung cancers based on a standardized-uptake value index. Eur J Cardiothorac Surg.

[2] Bechtel JJ, Petty TL, Saccomanno G (2000). Five year survival and later outcome of patients with X-ray occult lung cancer detected by sputum cytology. Lung Cancer.

[3] Lam S, MacAulay C, LeRiche JC (2000). Detection and localization of early lung cancer by fluorescence bronchoscopy. Cancer.

[4] Aihara H, Sumiyama K, Saito S (2009). Numerical analysis of the autofluorescence intensity of neoplastic and non-neoplastic colorectal lesions by using a novel videoendoscopy system. Gastrointest Endosc.

[5] Moghissi K, Dixon K, Stringer MR (2008). Current indications and future perspective of fluorescence bronchoscopy: a review study. Photodiagnosis Photodyn Ther.

[6] Lee P, Van den Berg RM., Lam S (2009). Color fluorescence ratio for detection of bronchial dysplasia and carcinoma *in situ*. Clin Cancer Res.

[7] Gibbs AR, Thunnissen FB (2001). Histological typing of lung and pleural tumors:third edition. J Clin Pathol.

[8] Jiang R, Ma CH, Zhu ZL (2014). Application of detecting cerebrospinal fluid circulating tumor cells in the diagnosis of meningeal metastasis of non-small cell lung cancer. Zhongguo Xian Dai Shen Jing Ji Bing Za Zhi.

[9] Ollier M Jr, Chamoux A, Naughton G (2014). Chest computed tomography screening for lung cancer in asbestos occupational exposure: a systematic review and meta-analysis. Chest.

[10] Frauenfelder T, Puhan MA, Lazor R (2014). Early detection of lung cancer: A statement from an expert panel of the Swiss University Hospitals on lung cancer screening. Respiration.

[11] Thakur A, Gao L, Ren H (2012). Descriptive data on cancerous lung lesions detected by auto-fluorescence bronchoscope: A five-year study. Ann Thorac Med.

[12] Divisi D, Di Tommaso S, De Vico A (2010). Early diagnosis of lung cancer using a SAFE-3000 autofluorescence bronchoscopy. Interac Cardiovasc Thorac Surg.

[13] Kennedy TC, Lam S, Hirsch FR (2001). Review of recent advances in fluorescence bronchoscopy in early localization of central airway lung cancer. Oncologist.

[14] Sun J, Garfield DH, Lam B (2011). The value of autofluorescence bronchoscopy combined with white light bronchoscopy compared with white light alone in the diagnosis of intraepithelial neoplasia and invasive lung cancer. J Thorac Oncol.

[15] Hanibuchi M, Yano S, Nishioka Y (2007). Autofluorescence bronchoscopy, a novel modality forthe early detection of bronchial premalignant and malignant lesions. J Med Inves.

[16] keda N, Honda H, Hayashi A (2006). Early detection of endobronchial lesions using newly developed videoendoscopy based autofluorescence bronchoscopy. Lung Cancer.

[17] Häussinger K, Becker H, Stanzel F (2005). Autofluorescence bronchoscopy with white light bronchoscopy compared with white light bronchoscopy alone for the detection of precancerous lesions: a European randomised controlled multicentre trial. Thorax.

[18] Hirsch FR, Prindiville SA, Miller YE (2001). Fluorescence versus white-light bronchoscopy for detection of preneoplastic lesions: a randomized study. J Natl Cancer Inst.

[19] Edell E, Lam S, Pass H (2009). Detection and localization of intraepithelial neoplasia and invasive carcinoma using fluorescence-reflectance bronchoscopy: an international, multicenter clinical trial. J Thorac Oncol.

[20] Ernst A, Simoff MJ, Mathur PN (2005). D-light autofluorescence in the detection of premalignant airway changes: a multicenter trial. Bronchol.

[21] Shen J, Ge SH (2000). YUV in Computer Systems. Ji Suan Ji Gong Cheng.

